# Association Between Low Tongue Pressure and Physical Abnormalities in Adolescent and Young Adult Women: A Cross-Sectional Study

**DOI:** 10.3390/children11121478

**Published:** 2024-12-02

**Authors:** Yuko Fujita, Shutaro Masuda, Tomohito Takeshima, Mai Fujimoto

**Affiliations:** 1 Division of Developmental Stomatognathic Function Science, Department of Health Promotion, Kyushu Dental University, Kitakyushu City 803-8580, Japan; r19masuda@fa.kyu-dent.ac.jp; 2 Takeshima Dental Office, Okinawa 904-2143, Japan; tomohiro1207045@gmail.com; 3 Mai Dental Clinic, Kitakyushu City 802-0056, Japan; maishikakodomoshika@mail.com

**Keywords:** tongue pressure, obesity, overweight, underweight, adolescent women

## Abstract

Background: We examined the factors associated with low tongue pressure to clarify its association with physical abnormalities in adolescent and young adult women. Methods: This study enrolled 92 women aged 10–20 years. Following the collection of anthropometric data, measurements were performed, including grip strength, maximum occlusal force, maximum tongue pressure, and masticatory performance. Based on the Rohrer index and body mass index (BMI), the participants were divided into normal weight, underweight, and overweight/obese groups. Low tongue pressure was defined as the bottom 20th percentile of maximum tongue pressure. After univariate analyses, factors associated with low tongue pressure were identified using multivariate binomial logistic regression analysis. Results: In the bivariate analysis, maximum tongue pressure was significantly positively correlated with grip strength (*r* = 0.407, *p* < 0.05). Binomial logistic regression analysis revealed that factors associated with low tongue pressure included being underweight (odds ratio [OR] = 7.451, *p* = 0.005, 95% confidence interval [CI] = 1.857–29.898), being overweight/obese (OR = 18.384, *p* = 0.004, 95% CI = 2.483–136.112), and having lower grip strength (OR = 0.864, *p* = 0.015, 95% CI = 0.768–0.972). Conclusions: Low tongue pressure is closely associated with being underweight, being overweight, and having lower grip strength among young women.

## 1. Introduction

The prevalence of obesity among adolescent girls has increased and become a social problem [1,2]. Adolescent obesity often continues into adulthood. Therefore, establishing measures to prevent and combat the childhood obesity epidemic is an urgent issue [3]. Conversely, more than one billion adolescent girls and women worldwide suffer from nutrient deficiencies and a number of related issues, including being underweight, with devastating consequences for their lives and wellbeing [4]. South Asia, West and Central Africa, and East and South Africa are the top three regions for underweight women, with 65% of all underweight women living in these regions in 2016 [5]. However, in 2022, Japan ranked first in the Asian region for the prevalence of underweight young women [6]. This rate is increasing, probably due to factors other than those in the above three regions (e.g., a desire to be thin) [7,8].

Sarcopenia is generally defined as a combination of reduced muscle mass, strength, and performance [9]. Being underweight leads to low lean body mass or low muscle mass in older adults [10] and is related to sarcopenia [11]. Recently, there has been a rising concern about “sarcopenic obesity”, which is a combination of obesity and decreased skeletal muscle mass and function [12]. One study suggested that muscle mass and strength in old age may reflect the peak muscle mass and strength gained during growth, as well as the rate of decline in peak muscle mass and strength [13]. Another study found that, in young adults, the prevalence of sarcopenia is higher in women than in men [14]. To prevent sarcopenia, girls should acquire as much peak muscle mass and strength as possible, along with ensuring proper nutritional intake and physical development. We believe that this also applies to perioral muscle strength. In recent years, maximum occlusal force and maximum tongue pressure have become typical evaluation items for assessing the strength of the perioral muscles, and these two muscle strengths are positively correlated with each other in girls [15].

The tongue is important for oral functions including chewing, swallowing, pronunciation, and occlusal formation [16,17,18,19]. A study on the relationship between oral function and sarcopenia in elderly outpatients suggested that sarcopenia may be associated with impaired tongue function [20]. Low tongue pressure generally refers to a condition in which elderly people are unable to exert sufficient tongue muscle strength when swallowing food and drink. It has been suggested that low tongue pressure is associated with choking in older adults; however, tongue pressure training may be effective in preventing dysphagia [21]. It has been reported that low tongue pressure also occurs in children and is associated with mouth breathing and malocclusion [17,22].

However, it is unclear whether being underweight or overweight is related to underdeveloped tongue pressure among young women. If we can clarify the relationship between physical abnormalities and underdeveloped tongue pressure in young women, we will be able to detect underdeveloped oral function from physical abnormalities earlier and intervene at an earlier stage.

The aims of this study were to identify factors related to low tongue pressure and to clarify the association between low tongue pressure and being underweight or overweight/obese in adolescent and young adult women. We hypothesized that low tongue pressure may be closely related to being underweight or obese in adolescent and young adult women.

## 2. Materials and Methods

### 2.1. Study Design and Participants

This study was a cross-sectional study that included participants enrolled after initial consultation at Kyushu Dental University Hospital between December 2022 and May 2023; patients were selected using a simple randomization technique. They provided informed consent before participation. Children aged 10–17 years old assented to participate, followed by parental consent. The Human Investigation Committee of Kyushu Dental University approved this study (Kitakyushu, Fukuoka, Japan; Approval Number 22-37).

For sample size calculations, we used the G*Power program (ver. 3.1.9.4 for Windows; available from the Heinrich Heine Universität Düsseldorf website) in a logistic regression model, obtaining an odds ratio (OR) of 6, an incidence rate of low tongue pressure in the normal weight group of 0.1, power of 0.80, and a probability of 0.05 for type I error for the null hypothesis. The analysis showed that the null hypothesis could be rejected with 74 participants. The inclusion criteria were age 10–20 years, good physical health, no problems affecting language comprehension, and no oral diseases. Participants with systemic illnesses resulting in impaired function of the tongue or perioral muscles, facial asymmetries that may have interfered with examinations, soft tissue disorders, temporomandibular joint disorders, abnormal tooth morphology, negative overjet, overbite, or those undergoing orthodontic treatment were excluded. Based on the power analysis, 92 women were enrolled in this study.

### 2.2. Anthropometric Measurements and Intraoral Examination

All participants had their height and weight measured in the examination room. Height was measured with a digital height gauge (AD-653; A&D, Tokyo, Japan) with an accuracy of ±0.1 cm, and weight was measured to an accuracy of 0.1 kg. Based on age-specific height and weight measurements, the Rohrer index (for participants aged 10–15 years) and the BMI (for participants aged 16–20 years) were calculated. Based on these scores, participants were categorized into three groups: underweight, normal weight, and overweight/obese [15,23].

In the intraoral examination, the decayed–missing–filled tooth (DMFT) index was calculated for all participants [24].

### 2.3. Grip Strength

Grip strength was measured using a portable grip strength meter (T-2288; Toei Light, Saitama, Japan). Grip strength measurement was taken twice, alternating between each hand with a 30 s interval. The greater value between the left and right sides was recorded as the grip strength in kilograms [25].

### 2.4. Maximum Occlusal Force

Maximum occlusal force was measured using a portable bite force meter (GM10; Nagano Keiki, Tokyo, Japan) equipped with a strain gauge wrapped in a plastic tube at the center of the bite element. The assessment was carried out with participants in a relaxed seated position. Participants were instructed to place the element on their upper first molars and then chew with maximal voluntary muscle force for about 3 s. A pressure gauge built into the element measured the bite force in kilonewtons (kN), which was displayed on an LCD screen. The maximum occlusal force measurement was taken bilaterally at a measurement interval of 30 s. The larger value between the left and right sides was recorded as the maximum occlusal force [26].

### 2.5. Maximum Tongue Pressure

A tongue pressure manometer (JMS; Hiroshima, Japan) was used for measurement of maximum tongue pressure. Participants were seated in a relaxed position, and the balloon was placed over the upper part of the tongue, in front of the palate. The participant closed their lips, and bit down on the hard ring between their upper and lower incisors. They were then instructed to raise their tongue and use maximal muscle strength to press the balloon against the roof of their mouth for approximately 7 s. A digital voltmeter connected to the manometer measured the maximum tongue pressure in kilopascals (kPa) [27]. Maximum tongue pressure measurements were taken twice with a 30 s measurement interval, and the greater value was recorded.

Based on the reported criteria for sarcopenia [28], participants in the lowest 20th percentile of maximum tongue pressure were defined as the low tongue pressure group. The remaining participants were assigned to the normal tongue pressure group.

### 2.6. Masticatory Performance

Masticatory performance was evaluated using the concentration of glucose dissolved in cylindrical gummy jelly (GLUCOLUMN; GC Corporation, Tokyo, Japan). The evaluator instructed the participants to masticate the gummy jelly for 20 s on their habitual chewing sides. After chewing, participants were asked to place 10 mL of distilled water in their mouths and spit the saliva, water, and gummy jelly pieces into a filtered cup. The glucose concentration (mg/dL) in the filtrate was read using a dedicated portable device and sensor chip (GLUCO SENSOR GS-II; GC) [26].

### 2.7. Measurements Reliability

All examinations of participants were performed repeatedly by the same single examiner, with a 30-min break between the examinations. The examiner was familiar with the measurement protocol. The reliability of the data generated was assessed via random error based on intraobserver reliability and quantified using the intraclass correlation coefficient (ICC); 0.8 ≤ ICC ≤ 1.0 indicated high reliability [29].

### 2.8. Data Analysis

The Shapiro–Wilk test was used to check the normality of the data. All measurements for each group are expressed as the mean ± standard deviation. Two-tailed *t* tests were used to compare means between two groups. Comparisons of the measured parameters among the three groups were performed using the Kruskal–Wallis test. Pearson’s bivariate correlation analysis was used to determine associations between age, anthropometric parameters, DMFT index, grip strength, and oral function. Comparisons of two categorical variables according to physique were made between participants with normal and low tongue pressures, using chi-squared tests. Residual analysis was performed when chi-square tests revealed significant associations between two variables. Binomial logistic regression analysis (conditional forward selection) was performed to identify the factors related to low tongue pressure. Independent variables with *p* values of less than 0.05 in the univariate analysis were included. Categorical variables were appropriately coded before they were entered into the model. Adjusted ORs and 95% confidence intervals (CIs) were calculated for the low tongue pressure group. All data were analyzed by the SPSS (ver. 23.0 for Windows; IBM Japan, Tokyo, Japan) and results were considered statistically significant at *p* < 0.05.

## 3. Results

The ICCs for all measurements ranged from 0.80 to 0.87, indicating high reliability.

Table 1 summarizes the mean age, anthropometric parameters, grip strength, and DMFT index according to physique. Of the participants, 22.8% were underweight, and 8.7% were overweight/obese. Grip strength was significantly lower in the underweight group than in the normal-weight group (*p* < 0.05).

**Figure 1:**
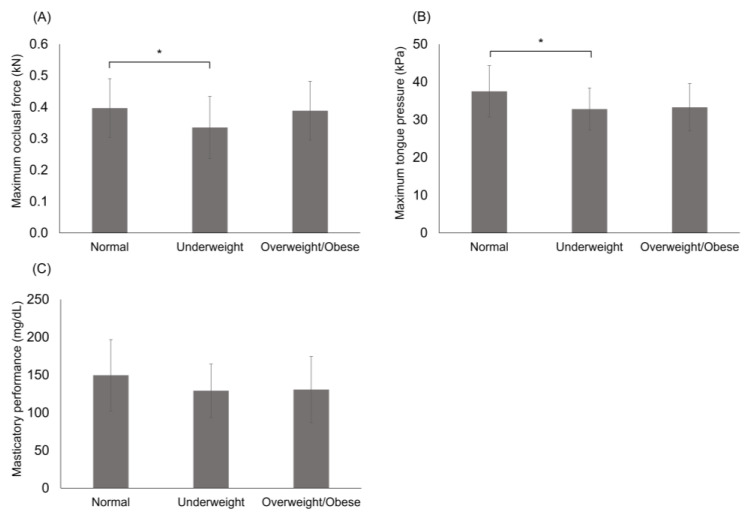
Comparison of oral function among three types of physique: (**A**) maximum occlusal force; (**B**) maximum tongue pressure; (**C**) masticatory performance. Data are expressed as means ± standard deviation. Differences between the groups were analyzed using a Kruskal–Wallis test; * *p* < 0.05.

Figure 1 shows the mean maximum occlusal force, maximum tongue pressure, and masticatory performance according to physique. The maximum occlusal force and tongue pressure in the underweight group were significantly lower than those in the normal weight group (both *p* < 0.05).

Table 2 compares age, height, body weight, Roher index, BMI, DMFT index, grip strength, and oral function between the low and normal tongue pressure groups. BMI and the DMFT index were significantly higher in the low tongue pressure group than in the normal tongue pressure group, whereas height, body weight, grip strength, maximum occlusal force, maximum tongue pressure, and masticatory performance were significantly lower in the low tongue pressure group (all *p* < 0.05).

Table 3 shows the data on low tongue pressure by physique, according to the chi-squared test followed by residual analysis. Participants with low tongue pressure were more likely to be underweight or overweight/obese (*p* = 0.001).

Table 4 shows the Pearson’s bivariate correlation coefficients between maximum tongue pressure and age and all measurements. Maximum tongue pressure was significantly positively correlated with grip strength (*r* = 0.407, *p* < 0.05) and maximum occlusal force (*r* = 0.410, *p* < 0.01).

Table 5 shows the factors associated with low tongue pressure, as revealed by binomial logistic regression. The factors related to low tongue pressure included being underweight (OR = 7.451, *p* = 0.005, 95% CI = 1.857–29.898) and being overweight/obese (OR = 18.384, *p* = 0.004, 95% CI = 2.483–136.112). For every 1 kg increase in grip strength, the odds of having low tongue pressure dropped by a factor of 0.864 (*p* = 0.015, 95% CI = 0.768–0.972).

## 4. Discussion

The goals of this study were to identify factors related to low tongue pressure and to clarify the association between low tongue pressure and being underweight or over-weight/obese in adolescent and young adult women. The most important finding in this study was that low tongue pressure in adolescent and young adult women was associated with both being underweight and being overweight/obese. In this study, binomial logistic regression analysis revealed that being overweight/obese was significantly associated with low tongue pressure in women aged 10–20 years old. A study of community-dwelling older women reported that sarcopenia was related to some physical difficulties in the obese state [30]. Our results showed that the grip strengths in the overweight/obese group and the normal-weight group were almost the same; consistent results were also found for maximum occlusal force. These results suggest that overweight or obesity in adolescent women does not significantly affect the instantaneous strength of skeletal and masseter muscles. However, the mean values of maximum tongue pressure and masticatory performance in the overweight/obese group were almost the same as those in the underweight group. In general, the tongue plays a very complex role in the process of chewing, firstly by changing its shape with precise timing during chewing to form the bolus of food and then by sending it from the oral cavity to the pharynx. Therefore, women with physique abnormalities may have problems in the development of chewing movements. A previous study reported that people with low chewing efficiency may be at risk of gaining body fat [31]. Another study reported that fewer chews and rapid eating decreased diet-induced thermogenesis in young adults [32]. These results suggest that underdeveloped tongue pressure may hinder the development of masticatory function and lead to obesity. A recent study reported that obese children tend to eat quickly and chew less [33]. Another possible explanation may be that underdeveloped masticatory function in overweight or obese individuals leads to the inadequate development of tongue pressure.

Our results revealed that underweight adolescents and young adult women had lower tongue pressure. A previous study has reported that malnutrition causes secondary sarcopenia in children as well as in the elderly [28]. Therefore, factors related to an individual’s general condition, poor dietary intake, nutritional status, or innate constitution may lead to low tongue pressure due to the underdevelopment of systemic muscle mass and strength. Moreover, if food intake is insufficient or the shape and hardness of the ingested food are not compatible with the developmental level of an individual’s mastication system, the development of perioral muscle mass, strength, and activity may be delayed, resulting in developmental failure of tongue pressure.

We demonstrated that grip strength was also a factor related to maximum tongue pressure. Currently, skeletal muscle strength is at the forefront of diagnosing sarcopenia, with grip strength being the preferred indicator [29]. A study of community-dwelling elderly people reported that decreased tongue pressure was related to an increased risk of malnutrition [34]. A decreased skeletal muscle mass index may predict decreased tongue pressure [35]. In our results, skeletal muscle strength (grip strength) was positively correlated with maximum tongue pressure. Maximum tongue pressure is an indirect measurement of tongue muscle strength, based on the pressure between the tongue and the palate; so, as with grip strength, participants with greater systemic muscle strength may have stronger tongue muscle. Therefore, sarcopenia may be related to undeveloped tongue pressure in women from adolescence through young adulthood. In addition, maximum occlusal force also showed a significant positive correlation with maximum tongue pressure in the bivariate analysis. Since maximum occlusal force has been reported to have a positive correlation with grip strength in girls [15], it may be indirectly associated with being underweight. However, in contrast to maximum occlusal force, tongue pressure was not necessarily associated with grip strength in overweight or obese women. These results suggest that systemic and oral sarcopenia, especially tongue sarcopenia, should be evaluated separately.

Recent studies have reported that both grip strength and maximum tongue pressure increase with age in women from their teens to their 20s [15,25]; however, a major difference between the two is that the rate of increase in maximum tongue pressure from ages 10 to 20 is lower than that in grip strength [15]. Our results showed a very low correlation between maximum tongue pressure and age. These results indicate that the growth patterns of grip strength and tongue pressure are not necessarily synchronized. Once women reach their 20s, their tongue muscle mass and strength may plateau and decline. Therefore, we need to detect and address undeveloped tongue pressure at an earlier stage. Additionally, we believe that for young women to obtain the highest possible peak tongue pressure, their parents should help them develop a strong physique and sufficient skeletal and perioral muscle mass and strength through proper exercise and feeding habits from an early age.

Our study has some limitations. First, the population in our study consisted of women aged 10–20 years old living a in specific region of Japan. Further studies with broader populations are needed to verify the generalizability of our findings. Second, only physically healthy women participated in the study. However, some of the included women may have been obese or underweight because of psychological factors. In the future, the causes of being obese and underweight should be included in the analysis.

## 5. Conclusions

This study demonstrated that being underweight, being overweight/obese, and having low grip strength were closely related to low tongue pressure in 10–20-year-old women. These results suggest that the physical condition of teenagers should be improved to aid tongue pressure development and prevent future decline in tongue pressure in women. When we examine and diagnose oral function in adolescent girls in our daily clinical practice, we must take into account the results of physical examinations.

## Figures and Tables

**Table 1:** Comparison of age, anthropometric parameters, DMFT index, and grip strength among the three groups according to physique.

	Normal (*n* = 63)	Underweight (*n* = 21)	Overweight/Obese (*n* = 8)
Age (years)	14.2 ± 2.9	13.5 ± 3.5	13.1 ± 2.4
Height (m)	1.48 ± 0.11	1.45 ± 0.10	1.49 ± 0.09
Body weight (kg)	43.0 ± 9.6	33.6 ± 6.8 *	51.3 ± 11.9 ^†^
Rohrer index	125.7 ± 18.5	108.0 ± 4.5 *	154.0 ± 13.9 *^†^
BMI	20.4 ± 1.6	17.3 ± 0.8 *	30.1 ± 5.7 *^†^
DMFT index	2.2 ± 2.3	6.0 ± 4.4 *	1.3 ± 2.4 ^†^
Grip strength (kg)	21.2 ± 7.0	16.3 ± 5.5 *	19.6 ± 9.2

Data are expressed as mean ± standard deviation. BMI, body mass index; DMFT, decayed, missing, and filled teeth. Differences between the groups were analyzed using the Kruskal–Wallis test; * *p* < 0.05 vs. normal group. † *p* < 0.05 vs. underweight group.

**Table 2:** Comparison of age, anthropometric parameters, DMFT index, and grip strength between the normal and low tongue pressure groups.

	Normal Tongue Pressure (*n* = 73)	Low Tongue Pressure (*n* = 19)
Age (years)	14.5 ± 10.2	13.0 ± 3.4
Height (m)	1.49 ± 0.10	1.41 ± 0.10 *
Body weight (kg)	43.3 ± 10.2	34.8 ± 8.7 *
Rohrer index	124.7 ± 21.6	122.1 ± 14.9
BMI	20.7 ± 2.5	22.6 ± 9.4 *
DMFT index	2.3 ± 2.3	5.4 ± 5.2 *
Grip strength (kg)	21.2 ± 6.8	14.8 ± 6.1 *
Maximum occlusal force (kN)	0.4 ± 0.1	0.3 ± 0.1 *
Maximum tongue pressure (kPa)	38.3 ± 5.6	27.4 ± 2.6 *
Masticatory performance (mg/dL)	150.9 ± 45.7	113.9 ± 27.6 *

Data are expressed as mean ± standard deviation. BMI, body mass index; DMFT, decayed, missing, and filled teeth. Differences between normal and low tongue pressure groups were analyzed using a two-tailed *t* test. * *p* < 0.05 vs. normal tongue pressure group.

**Table 3:** Comparison of participants with low tongue pressure according to physique. Chi-square test.

	Normal Tongue Pressure (%)	Low Tongue Pressure (%)	*χ* ^2^	*p*-Value
Normal	57 (78.1)	6 (31.6)		
Underweight	12 (16.4)	9 (47.4)		
Overweight/obese	4 (5.5)	4 (21.1)		
			15.284	<0.001

**Table 4:** Peason’s correlation coefficients between the maximum tongue pressure and age, anthropometry, DMFT index, grip strength, and other oral function.

	Age	Height	Body Weight	Rohrer Index	BMI	DMFT Index	Grip Strength	Maximum Occlusal Force	Masticatory Performance
Maximum tongue pressure	0.290 **	0.381 **	0.373 **	−0.163	−0.038	−0.271 **	0.407 *	0.410 **	0.286 **

BMI, body mass index; DMFT, decayed, missing, and filled teeth. * *p* < 0.05, ** *p* < 0.01.

**Table 5:** Factors related to low tongue pressure according to binary logistic regression analysis.

Independent Variables	Category	Adjusted Odds Ratio (95% CI)	*p*-Value
Physique	Normal	1	―
	Underweight	7.451 (1.857–29.898)	0.005
	Overweight/obese	18.384 (2.483–136.112)	0.004
Grip strength (per 1 kg increase)		0.864 (0.768–0.972)	0.015

Forward selection (conditional) method; 2 Log likelihood = 62.034; Hosmer and Lemeshow test: *χ*^2^ = 8.368, *p* = 0.398; Cox–Snell R^2^ = 0.246; Nagelkerke R^2^ = 0.364. CI, confidence interval.

## Data Availability

Data are contained within the manuscript.
